# Patterns of Deep-Sea Genetic Connectivity in the New Zealand Region: Implications for Management of Benthic Ecosystems

**DOI:** 10.1371/journal.pone.0049474

**Published:** 2012-11-21

**Authors:** Eleanor K. Bors, Ashley A. Rowden, Elizabeth W. Maas, Malcolm R. Clark, Timothy M. Shank

**Affiliations:** 1 Biology Department, Woods Hole Oceanographic Institution, Woods Hole, Massachusetts, United States of America; 2 National Institute of Water and Atmospheric Research, Greta Point, Wellington, New Zealand; University of Glasgow, United Kingdom

## Abstract

Patterns of genetic connectivity are increasingly considered in the design of marine protected areas (MPAs) in both shallow and deep water. In the New Zealand Exclusive Economic Zone (EEZ), deep-sea communities at upper bathyal depths (<2000 m) are vulnerable to anthropogenic disturbance from fishing and potential mining operations. Currently, patterns of genetic connectivity among deep-sea populations throughout New Zealand’s EEZ are not well understood. Using the mitochondrial *Cytochrome Oxidase I* and *16S rRNA* genes as genetic markers, this study aimed to elucidate patterns of genetic connectivity among populations of two common benthic invertebrates with contrasting life history strategies. Populations of the squat lobster *Munida gracilis* and the polychaete *Hyalinoecia longibranchiata* were sampled from continental slope, seamount, and offshore rise habitats on the Chatham Rise, Hikurangi Margin, and Challenger Plateau. For the polychaete, significant population structure was detected among distinct populations on the Chatham Rise, the Hikurangi Margin, and the Challenger Plateau. Significant genetic differences existed between slope and seamount populations on the Hikurangi Margin, as did evidence of population differentiation between the northeast and southwest parts of the Chatham Rise. In contrast, no significant population structure was detected across the study area for the squat lobster. Patterns of genetic connectivity in *Hyalinoecia longibranchiata* are likely influenced by a number of factors including current regimes that operate on varying spatial and temporal scales to produce potential barriers to dispersal. The striking difference in population structure between species can be attributed to differences in life history strategies. The results of this study are discussed in the context of existing conservation areas that are intended to manage anthropogenic threats to deep-sea benthic communities in the New Zealand region.

## Introduction

Current anthropogenic pressures on the marine environment, including the deep sea, are unprecedented [1], [2], [3], [4]. As the human footprint in the oceans increases, international agreements like the UN Convention on Biodiversity have spurred the creation of several national biodiversity task forces that acknowledge the importance of marine protected areas (MPAs) – i.e., any area of the marine environment that has been reserved by laws or regulations to provide lasting protection to part or all of the natural or cultural resources therein (e.g., [5], [6]). However, the creation of such areas is often a political, social, and scientific challenge (e.g., [7]).

Genetic connectivity has recently come to the fore as a major scientific component of sound MPA design in both shallow and deep-sea environments (e.g., [8], [9], [10], [11]). Genetic connectivity, or “the dispersal, survival, and reproduction of migrants, so that they contribute to the local gene pool” [12], examines temporal and spatial aspects of population genetics in order to infer the degree of genetic exchange among populations. The theoretical optimization of MPA design arises from understanding the sources and sinks of marine populations so that MPAs can protect sites that will export individuals to other areas, thus increasing the net benefit of the MPA [8]. Genetic connectivity research generally focuses on patterns of population structure within a geographic area and the factors that could cause such population structure to arise.

Ranking as the sixth largest globally, the New Zealand Exclusive Economic Zone (EEZ) is one of the most topographically diverse seafloor environments in the world [13]. Benthic habitats are provided by a continental slope with canyons and cold seeps, while further off shore there are numerous plateau, rises, troughs, ridges, basins, seamounts (many with hydrothermal vents), as well as two ocean trenches [14]. The New Zealand EEZ supports rich biodiversity [15], economically important and well-established fisheries [16], and provides for other extractive industries, including hydrocarbon and mineral mining [17], [18]. Of the many species commercially targeted by New Zealand’s fisheries, just ten deep-water species comprise 70% of the total catch volume [19], and bottom trawling occurs at depths down to 1500 m throughout the EEZ (e.g., [20]). The physical disturbance from trawl gear can have profound effects on deep-sea benthic communities, particularly on seamounts [21], where communities are thought to be more susceptible to disturbance from trawling because the fauna are less adapted to frequent natural disturbances and have life history traits that make them particularly vulnerable to fishing [22], [23]. Bottom trawling on non-seamount habitat is extensive in the New Zealand EEZ, with areas of the seabed on the Chatham Rise having been subjected to tens of thousands of trawls between 1989–2005 (e.g., fishing statistical area, Figure 17 from [20]). While the impact of fishing on benthic communities at non-seamount habitats is generally unknown in the New Zealand EEZ, invertebrate by-catch studies have indicated a likely disturbance to soft sediment communities on the continental margin slope and certain areas of the Chatham Rise [24], [25]. In addition to fishing operations, interest in mining has increased. Seafloor areas of the Chatham Rise contain significant deposits of phosphorite nodules [18] and several companies have been granted exploratory permits [26].

Currently, there is no legislation that allows for the creation of marine reserves (defined by current New Zealand law as MPAs in which only scientific uses are allowed) in the New Zealand EEZ (i.e., outside of the 12 nautical mile territorial seas), limiting the tools available for management of human activities in New Zealand’s deep sea. There are areas closed to bottom trawling that include specific seamounts [27] and fishing industry-created Benthic Protection Areas (BPAs) [28]. But, activities such as mid-water trawling and mining are allowed at closed seamounts and in BPAs, a fact which has raised the concern that this specific type of closure does not fulfill biodiversity goals for New Zealand’s EEZ. To date, only one published study has addressed the placement of the BPAs [29], despite their imminent 2013 review.

Most population genetic studies in the New Zealand region have been carried out in coastal waters (e.g., [30], [31], [32], [33]), with relatively few studies of deep-water species. Smith *et al*. (2004) [34] examined connectivity of hydrothermal vent mussels between two seamounts in the Kermadec Arc, north of New Zealand. Allozyme loci revealed unexpected levels of heterogeneity between the seamount populations despite only 50 km of separation. The authors attribute the finding to localized current regimes promoting isolation of these populations. Using mitochondrial *Cytochrome Oxidase I* (*COI*) data, Kojima *et al.* (2006) [35] demonstrated that the population of *Lamellibrachia juni* tubeworms at Brothers seamount in the Kermadec Volcanic Arc contains two distinct genetic groups, one of which was phylogenetically related to samples from the TOTO caldera in the Mariana Volcanic Arc. These studies show how complex patterns can exist over various spatial scales. Similarly, genetic investigation of the *Internal Transcribed Spacer* regions 1 and 2, *COI* and *16S rRNA* genes in populations of the coral *Desmophyllum dianthus* from Chile, New Zealand, and Australia revealed greater variation between populations at different depths within a region than between populations at the same depth in two different regions [36]. Corals in the New Zealand mid-depth stratum were more similar to corals in a mid-depth stratum in Australia than to corals in shallower water in New Zealand, and geographic genetic structure was not observed within the New Zealand region by this study [36]. A study of Keratoisidinae bamboo corals in the Western Pacific using the INDEL#2 region of *16S rRNA* and a non-coding mitochondrial marker also found no genetic structure in the New Zealand region–which may present an accurate evolutionary pattern or may be the result of using evolutionarily conserved genetic markers that can be slow to change over time [37].

**Figure 1:**
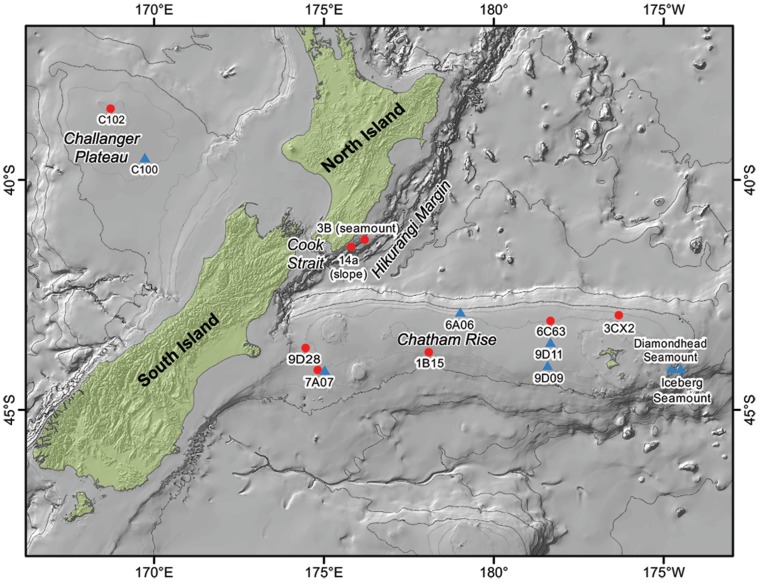
The location of the study area, including the North and South Islands of New Zealand (landmasses are in green), the Challenger Plateau, Hikurangi Margin, and Chatham Rise. Red circles mark sites from which *Munida gracilis* were collected; blue triangles mark sites from which *Hyalinoecia longibranchiata* were collected. Sites are labeled with their original site names. Samples were selected within a depth band of 400–800 m with *Munida gracilis* between 421 m and 634 m, and *Hyalinoecia longibranchiata* between 478 m to 746 m. The depth of each site is listed in Table 1.

The present study aims to elucidate patterns of genetic connectivity among populations of benthic invertebrates found at three different deep-sea regions–a prominent rise, an adjacent slope margin, and a nearby plateau–and to consider the implications of the observed patterns for management decisions. The three study regions were the Chatham Rise, the Hikurangi Margin, and the Challenger Plateau (Figure 1). The Chatham Rise is a submerged feature that extends about 800 kilometers to the east of the South Island of New Zealand. There are numerous seamounts on the Rise, including the Graveyard Seamount cluster on the northern flank and the Andes Seamount cluster on the southeastern edge of the rise [38]. The Subtropical Front (STF), a convergence zone between the subtropical and subantarctic water masses, extends west to east along the rise at the confluence of the East Cape Current and the Southland Current [39]. To the northwest of the Chatham Rise is Cook Strait, which separates the North and South Islands of New Zealand. The Hikurangi Margin is at the eastern opening of Cook Strait. Small seamounts are found across the slope of the Margin, which is also incised with numerous canyons. The Challenger Plateau extends off the continental margin to the west side of the Cook Strait.

**Table 1:** Sites and collected samples included in this study.

Site Name	Cruise	Location	Latitude	Longitude	Date	Depth	Species
9D11	TAN0705	Chatham Rise	43.6287 S	178.3664 W	18-Apr-07	421	MG
9D09	TAN0705	Chatham Rise	44.0682 S	178.3295 W	18-Apr-07	450	MG
9D28	TAN0705	Chatham Rise	43.7257 S	174.458 E	27-Apr-07	550	HL
6A06	TAN0705	Chatham Rise	42.9935 S	178.9992 E	24-Apr-07	530	MG
7A07	TAN0705	Chatham Rise	44.1358 S	174.8438 E	4-Apr-07	518	HL, MG
3CX2	TAN0705	Chatham Rise	42.9988 S	176.3483 W	16-Apr-07	658	HL
1B15	TAN0705	Chatham Rise	43.8085 S	178.1173 E	7-Apr-07	497	HL
6C63	TAN0705	Chatham Rise	43.1575 S	178.3097 W	17-Apr-07	478	HL
C102	TAN0707	Challenger Plateau	38.3872 S	168.7397 E	29-May-07	482	HL
C100	TAN0707	Challenger Plateau	39.5437 S	169.7145 E	4-Jun-07	634	MG
Iceberg Seamount	TAN0905	Andes Seamounts	44.1582 S	174.555 W	28-Jun-09	551	MG
DiamondheadSeamount	TAN0905	Andes Seamounts	44.1473 S	174.6900 W	26-Jun-09	520	MG
14a (slope)	TAN1004	Hikurangi Margin	41.5195 S	175.8068 E	19-Apr-10	746	HL
3B (seamount)	TAN1004	Hikurangi Margin	41.3368 S	176.182 E	21-Apr-10	730	HL

We focus on two benthic invertebrates–the squat lobster *Munida gracilis* [40] and the onuphid, or “quill,” worm *Hyalinoecia longibranchiata* [41]. Both species are abundant and widely distributed in the New Zealand region, existing throughout our study area (K. Schnabel, pers comm.), [42]. These species have strongly contrasting inferred modes of reproduction and dispersal. There is likely a long pelagic larval duration via planktonic dispersal in *M. gracilis*, as is typical for many *Munida* species [43]. Development is non-planktotrophic in onuphids [44], with some *Hyalinoecia* species having incubated embryos [45], [46]. These species are used here to represent commonly occurring benthic organisms with contrasting life history strategies.

Our study of the population genetics of these two species aimed to address fundamental questions regarding connectivity of the deep benthos among some of the prominent geomorphic features in the New Zealand EEZ: (1) Is there regional genetic structure across the study area and if so, can this structure be explained by factors known to affect genetic connectivity (e.g., currents, geographic distribution, topography, habitat availability)?; (2) Is there significant genetic structure within the three regions? For example, is there a difference among populations that are found in different habitats but are geographically close together? Is there significant genetic structure between populations on the north and south flanks of the Chatham Rise, potentially influenced by the presence of the Subtropical Front?; (3) Do the inferred life history strategies correlate with the observed patterns of genetic connectivity?; and (4) What implications do the patterns of genetic population connectivity between species and among sample sites, habitats, and regions have for Marine Protected Area design and the efficacy of the current Benthic Protection Areas?

**Table 2:** Intra-population mt*COI* diversity statistics for the squat lobster, *Munida gracilis*.

Site (region)	n	S	h	Hd	π
**C100 (CP)**	6	6	4	0.8	0.00418
**7A07 (CR)**	4	11	4	1.00000	0.01109
**6A06 (CR)**	8	12	7	0.96429	0.00808
**9D11 (CR)**	8	13	8	1.00000	0.00727
**9D09 (CR)**	8	12	8	1.00000	0.00693
**Iceberg Seamount (CR)**	10	19	9	0.97778	0.00837
**Diamondhead Seamount (CR)**	8	6	7	0.96429	0.00395
**Total**	52	47	36	0.96003	0.00691

Regions are designated as CP for Challenger Plateau and CR for Chatham Rise. *n* is the total number of individuals sampled for a site, S is the number of polymorphic nucleotide sites in the sequence, h is the number of haplotypes represented at the site, Hd is haplotype diversity, and pi is nucleotide diversity.

**Table 3:** Intra-population *16S* diversity statistics for the quill worm, *Hyalinoecia longibranchiata*.

Site (region)	n	S	h	Hd	π
**C102 (CP)**	12	6	7	0.90909	0.00294
**3B (HM)**	6	3	2	0.53333	0.00235
**14a (HM)**	10	4	3	0.51111	0.00187
**9D28 (CR)**	8	3	4	0.64286	0.00110
**7A07 (CR)**	8	5	5	0.85714	0.00210
**1B15 (CR)**	6	1	2	0.33333	0.00049
**6C63 (CR**	5	2	3	0.80000	0.00147
**3XC2(CR)**	6	1	2	0.33333	0.00049
**Total**	61	19	19	0.86011	0.00433

Regions are designated as CP for Challenger Plateau and CR for Chatham Rise. *n* is the total number of individuals sampled for a site, S is the number of polymorphic nucleotide sites in the sequence, h is the number of haplotypes represented at the site, Hd is haplotype diversity, and pi is nucleotide diversity.

**Table 4:** Intra-population mt*COI* diversity statistics for the quill worm, *Hyalinoecia longibranchiata*.

Site (region)	n	S	h	Hd	π
**C102 (CP)**	9	6	3	0.55556	0.00413
**3B (HM)**	7	5	2	0.47619	0.00454
**14a (HM)**	8	12	4	0.78571	0.00988
**9D28 (CR)**	7	5	5	0.85714	0.00309
**7A07 (CR)**	8	5	5	0.85714	0.00354
**1B15 (CR)**	7	5	3	0.66667	0.00309
**6C63 (CR)**	6	4	3	0.73333	0.00331
**3XC2 (CR)**	6	2	3	0.73333	0.00178
**Total**	58	30	20	0.92801	0.01381

The regions are designated as CP for Challenger Plateau and CR for Chatham Rise.*n* is the total number of individuals sampled for a site, S is the number of polymorphic nucleotide sites in the sequence, h is the number of haplotypes represented at the site, Hd is haplotype diversity, and pi is the is nucleotide diversity.

## Methods

### Sample Collection and Study Sites

Populations of *Hyalinoecia longibranchiata* and *Munida gracilis* were collected during four research cruises onboard the R/V *Tangaroa*: TAN0705 (Chatham Rise, March 31^st^ to April 29^th^ 2007), TAN0707 (Challenger Plateau, May 28^th^ to June 8^th^ 2007), TAN0905 (Andes and Graveyard Seamounts, June 12^th^ to June 30^th^ 2009), and TAN1004 (Hikurangi Margin including the slope and seamounts near the eastern side of the Cook Strait, April 14^th^ to April 29^th^ 2010). All necessary permits were obtained for the described field studies. The specimens used in this study were taken from samples collected or obtained by New Zealand’s National Institute of Water and Atmospheric Research (NIWA) under a “Special Permit (421)” issued by the New Zealand Ministry of Fisheries for the taking of fish, aquatic life, and seaweed for the purposes of education and investigative research. Samples were collected using NIWA’s epibenthic “seamount sled” (overall size 150 cm long, 50 cm high, and 100 cm wide; macro-invertebrates retained in a 30 mm stretched mesh size net that was covered in an anti-chaffing net of 100 mm stretched mesh size), a hyperbenthic “Brenke” sled, and a beam trawl. Upon collection, *M. gracilis* and *H. longibranchiata* specimens were preserved in ethanol, except for 29 *H. longibranchiata* individuals that were frozen upon collection. All specimens are stored in the NIWA Invertebrate Collection (NIC).

**Figure 2:**
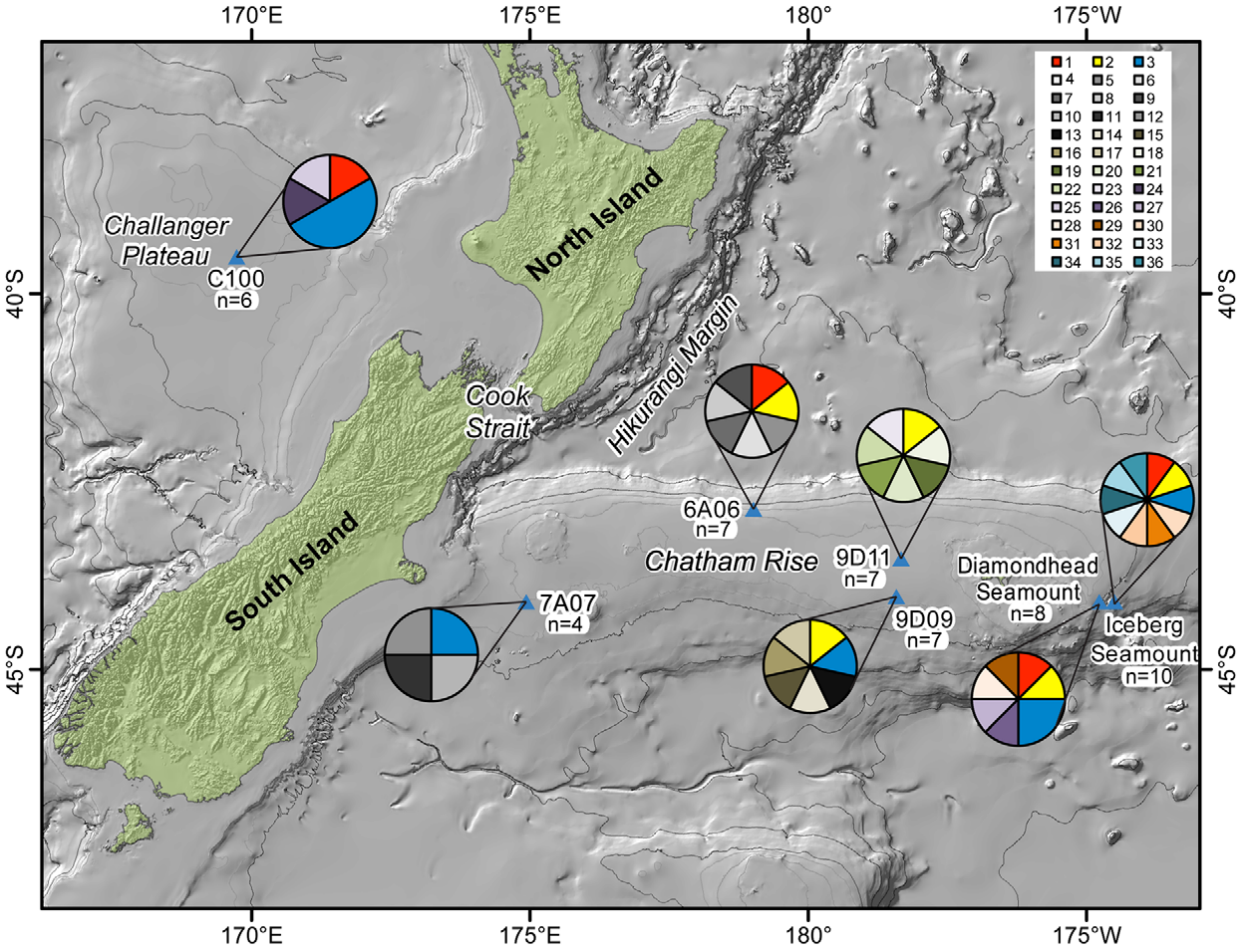
Distribution of *COI* haplotypes across the study area for *Munida gracilis*. The map shows the location of the study sites with pie charts indicating the haplotype composition of the population from that site. Each color represents a haplotype with Red, Blue, and Yellow representing the three shared haplotypes that are found across the study area. Shades of grey and other muted colors represent unique haplotypes. Sample size for each site is indicated.

Due to limitations in the number of sampled individuals and resources available to this study, not all sample sites for which there were specimens in the NIC could be used in this study. In order to avoid confounding geographic site comparisons with depth variability, populations (the combined individual samples) from sites were selected from a restricted depth range (400–800 m). Sites did not straddle a previously identified depth disjunction in population structure between populations at <600 and >1000 m [36]. Individuals of *H. longibranchiata* were sampled from 478 m to 746 m and *M. gracilis* from 421 m to 634 m depth. To explore the role of geomorphological habitat types as a factor for structuring connectivity, we identified sites that spanned habitat types in the three regions (e.g., seamount and slope). When possible, sites with samples for both species were used.

**Figure 3:**
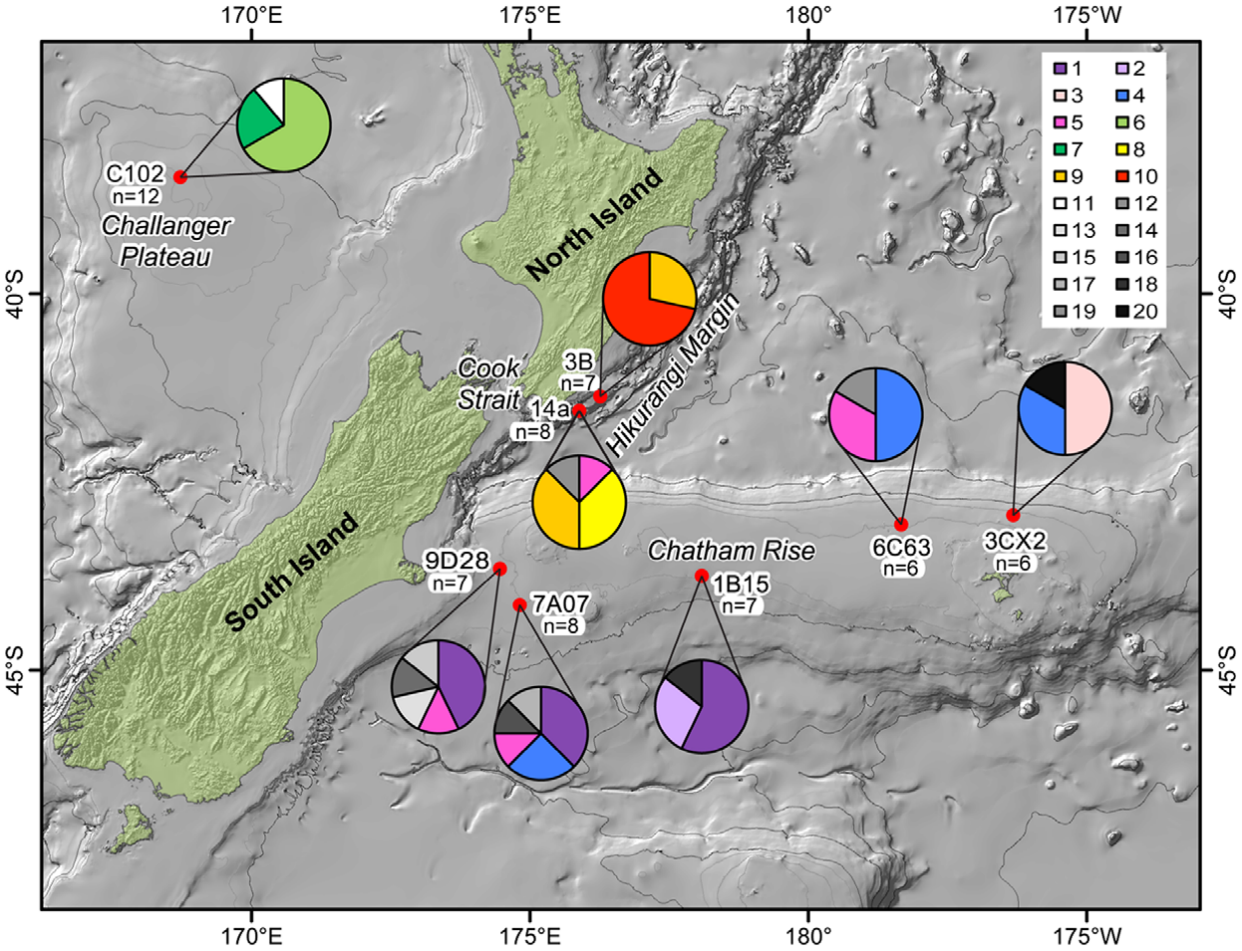
Distribution of *COI* haplotypes across the study area for *Hyalinoecia longibranchiata*. The location of the study sites with pie charts indicate the haplotype composition of the population from that site. Each color represents a shared haplotype. White, black, and shades of grey represent unique haplotypes. Sample sizes for each site are indicated.

**Figure 4:**
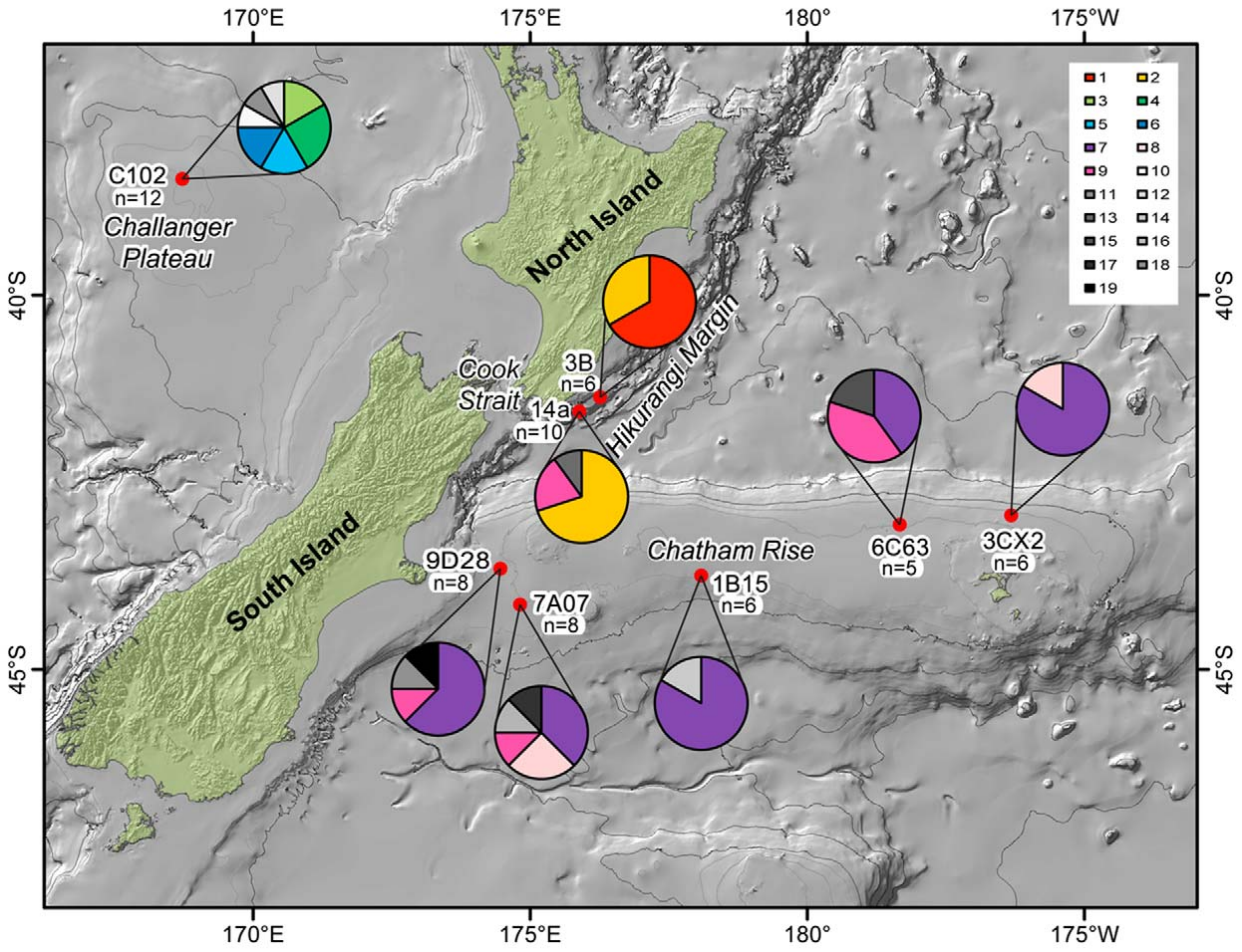
Distribution of *16S* haplotypes across the study area for *Hyalinoecia longibranchiata*. The map shows the location of the study sites with pie charts indicating the haplotype composition of the populations from that site. Each color represents a shared haplotype. White, black, and shades of grey represent unique haplotypes. Sample size for each site is indicated.

The study sites (i.e., sampled populations) are presented in Table 1 and Figure 1. One population for each species was identified on the Challenger Plateau: “C102” for *H. longibranchiata* and “C100” for *M. gracilis*. Two sites from the Hikurangi Margin were used for *H. longibranchiata*: “14a,” a slope habitat site and “3B,” a seamount site. On the Chatham Rise, five sites were used for *H. longibranchiata*: “7A07,” “9D28,” and “1B15” on the southwest part of the rise, and “6C63” and “3CX2” on the northeast part of the rise. Six sites on the Chatham Rise were identified for *M. gracilis*: “7A07” in the southwest, “6A06” centrally located on the northern flank of the rise, “9D11” and “9D09” located in the south-central region of the rise, and “Iceberg Seamount” and “Diamondhead Seamount” of the Andes Seamount cluster at the eastern end of the rise.

**Figure 5:**
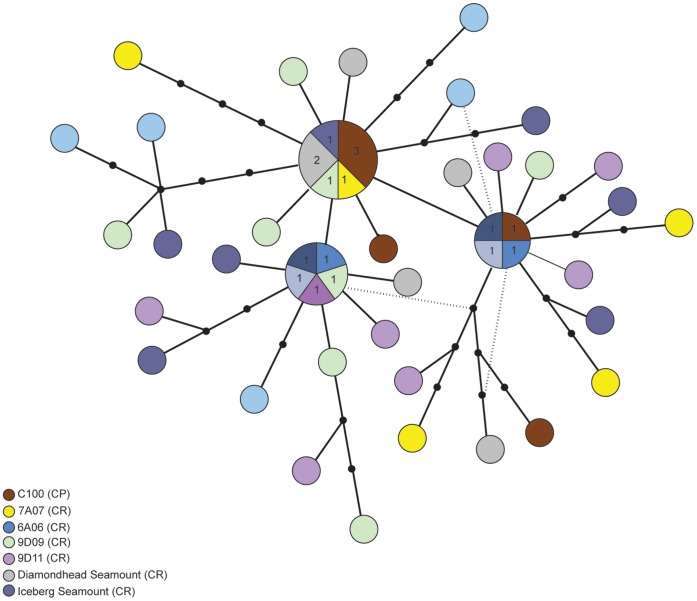
TCS haplotype network for *Munida gracilis*, *COI* sequences. Each circle represents an observed haplotype and the circles are proportional to the number of individuals sampled with that haplotype. Each color indicates a sampling site and when a haplotype was present at multiple sites, a pie chart indicates the proportions with absolute numbers appearing in text in the pie chart. Each line connecting colored circles represents a single nucleotide sequence change. Lines with small black circles indicate interior haplotypes not found in the dataset (multiple nucleotide changes between sampled haplotypes).

### DNA Extraction, Polymerase Chain Reaction, and Sequencing

Mid-section muscular tissue from *H. longibranchiata* and leg tissue from *M. gracilis* were sub-sampled for genomic DNA (gDNA) extraction. To increase gDNA yield, many ethanol-preserved samples (n = 61) were soaked for 24 hours in a buffer containing 500 mM Tris-HCL (pH8), 20 mM EDTA, and 10 mM NaCl before extraction [47]. Genomic DNA was extracted using the QIAGEN DNeasy Blood and Tissue extraction kit following the manufacturer’s instructions (Qiagen GmbH, Germany) with a final elution into 25 to 200 µl of RNAase/DNAse free H_2_O (Invitrogen Ltd, New Zealand), depending on the condition of the original tissue sample. For samples with poor tissue quality due to disintegration in ethanol, elution occurred in smaller volumes of water in order to achieve a higher concentration of gDNA. Genomic DNA was quantified using Quant-iT PicoGreen DNA quantification kit according to the manufacturer’s instructions (Invitrogren Ltd, New Zealand), and working stocks of DNA (approximately 10 ng/µl) were stored at 4°C for up to six months prior to use.

**Figure 6:**
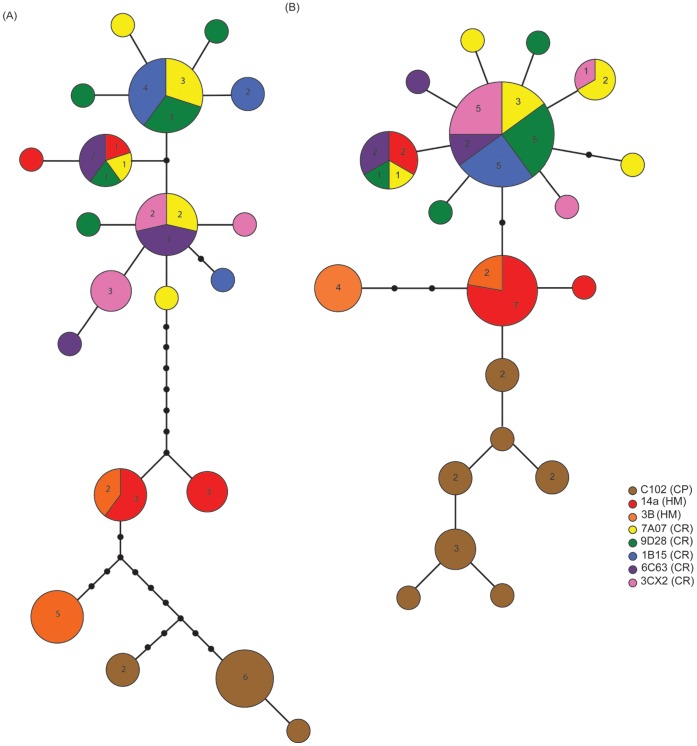
TCS haplotype networks for *Hyalinoecia longibranchiata*. Part (A) shows results for *COI* sequences and part (B) shows results for *16S* sequences. Each circle represents an observed haplotype and the circles are proportional to the number of individuals sampled with that haplotype. Each color indicates a sampling site and when a haplotype was present at multiple sites, a pie chart indicates the proportions with absolute numbers appearing in text in the pie chart. Each line connecting colored circles represents a single nucleotide sequence change. Lines with small black circles indicate interior haplotypes not found in the dataset (multiple nucleotide changes between sampled haplotypes).

For both target species, a fragment of the mitochondrial *COI* gene was amplified using universal primers [48]. The *COI* gene was amplified using iProof High-Fidelity DNA Polymerase Master Mix (Bio-Rad Ltd, Australia), using 1–5 µl of gDNA and primer concentrations of 0.2 mM each. A subset of reactions was trialed with HOT FIREPol Master Mix with 1.5 mM MgCl_2_ (Solis BioDyne) in an unsuccessful attempt to increase PCR yield. A “touch-up” PCR profile was used to eliminate non-specific binding. The profile used for *COI* consisted of denaturing at 98°C for 2 minutes followed by 10 cycles of denaturing at 98°C for 10 seconds, annealing at 49°C incrementally raising to 54°C for 30 seconds, and extension at 72°C for 30 seconds; followed by twenty cycles of denaturing at 98°C for 10 seconds, annealing at 54°C for 30 seconds, and extension at 72°C for 30 seconds; with a final extension step of 72°C for 7 minutes in a 2700 Applied Biosystems PCR machine. For some samples with low gDNA concentrations, an extra ten cycles (for a total of 30 cycles) were added in the final PCR profile.

**Table 5:** Pairwise Fst values between populations of the squat lobster, *Munida gracilis*, using a fragment of the *COI* gene.

	C100	7A07	6A06	9D11	9D09	Diamondhead Seamount	Iceberg Seamount
**C100**		–	–	–	–	–	–
**7A07**	0.04135		–	–	–	–	–
**6A06**	0.02383	0.03294		–	–	–	–
**9D11**	0.03655	0.04033	0.04692		–	–	–
**9D09**	−0.02666	0.02273	−0.0309	0.01299		–	–
**Diamondhead Seamount**	−0.04481	0.0536	−0.0064	−0.00304	−0.03896		–
**Iceberg Seamount**	−0.05198	−0.00004	−0.03325	−0.00142	−0.05346	−0.05184	

Above the diagonal indicates ranges of p-values. The “−” denotes a p>0.05. The “*” denotes a p<0.05. The “**” denotes a p<0.01. The “***” denotes a p<0.001.

**Table 6:** Pairwise Fst Values between populations of the quill worm, *Hyalinoecia longibranchiata,* using a fragment of the *16S* gene.

	C102	3B	14a	9D28	7A07	1B15	6C63	3CX2
**C102**		***	***	***	***	***	***	***
**3B**	0.57829		**	***	***	***	***	***
**14a**	0.564	0.47989		**	***	***	*	***
**9D28**	0.73021	0.7446	0.53759		–	–	–	–
**7A07**	0.69463	0.68352	0.484	0.00408		–	–	–
**1B15**	0.73454	0.768	0.56923	−0.01659	0.01118		–	–
**6C63**	0.70138	0.71154	0.50162	0.01202	0.01408	0.15141		*
**3CX2**	0.74672	0.78462	0.58732	−0.0084	−0.05466	0.00201	0.17647	

Above the diagonal indicates ranges of p-values. The “−” denotes a p>0.05. The “*” denotes a p<0.05. The “**” denotes a p<0.01. The “***” denotes a p<0.001.

Primers used for *H. longibranchiata 16S* gene amplification were from Zanol *et al.* (2010) [49]. The *16S* gene was amplified using a “touch-down” method as described in Zanol *et al.* (2010) [49]. iProof High-Fidelity DNA Polymerase Master Mix (Bio-Rad Ltd, Australia) was used with 1–5 µl of gDNA and primer concentrations of 0.2 mM each. A portion of *16S* was sequenced for a small subset of *M. gracilis* samples (n = 7); however, the portion of the genetic marker that we were able to sequence exhibited no variation among the sequenced individuals. The same was true for a small set of *Internal Transcribed Spacer Region* sequences (n = 12) generated for *H. longibranchiata*.

**Table 7:** Pairiwse Fst values between populations of the quill worm, *Hyalinoecia longibranchiata,* using a fragment of the *COI* gene.

	C102	3B	14a	9D28	7A07	1B15	6C63	3XC2
**C102**		***	***	***	***	***	***	***
**3B**	0.76723		**	***	***	***	***	***
**14a**	0.66914	0.38421		***	**	***	***	***
**9D28**	0.84878	0.81818	0.58117		–	–	**	***
**7A07**	0.83886	0.80383	0.56703	−0.00474		–	–	**
**1B15**	0.85298	0.82278	0.59817	−0.0303	0.06264		***	**
**6C63**	0.84071	0.80985	0.54635	0.31089	0.10061	0.38353		–
**3XC2**	0.86515	0.84923	0.62322	0.53833	0.33767	0.5605	0.11111	

Above the diagonal indicates ranges of p-values. The “−” denotes a p>0.05. The “*” denotes a p<0.05. The “**” denotes a p<0.01. The “***” denotes a p<0.001.

PCR amplification was assessed using gel electrophoresis and Quant-iT PicoGreen DNA quantification kit according to the manufacturer’s instructions (Invitrogren Ltd, New Zealand). PCR products of the correct size were purified using either a Zymogenetics PCR purification kit (Zymogentics D4013) or a Qiagen PCR purification kit (Qiagen GmbH, Germany) and eluted in DNA/RNAase-free water. Purified PCR reactions of approximately 10 ng were shipped to Macrogen, Inc. for sequencing.

**Table 8:** *16S* AMOVA results for *Hyalinoecia longibranchiata*.

Test	Source of variation	df	SS	Var. comp.	% V	P value
Three Regions	among regional groups	2	49.641	1.24887 Va	63.54	0.00782±0.00280
	among populations within regional groups	5	7.231	0.12367 Vb	6.29	0.00000±0.00000
	within populations	54	32.015	0.59288 Vc	30.17	0.00000±0.00000
Margin v. Rise	among regional groups	1	18.894	0.79467 Va	55.88	0.04399±0.00714
	among populations within regional groups	5	7.231	0.13876 Vb	9.76	0.00293±0.00164
	within populations	43	21.015	0.48873 Vc	34.37	0.00000±0.00000
NE v. SW CR	among regional groups	1	0.195	−0.02046 Va	−5.27	1.00000±0.00000
	among populations within regional groups	3	1.548	0.01853 Vb	4.77	0.10459±0.00793
	within populations	29	11.315	0.39019 Vc	100.5	0.24829±0.01653

**Table 9:** *COI* AMOVA results for *Hyalinoecia longibranchiata*.

Test	Source of variation	df	SS	Var. comp.	% V	P value
Three regions	among regional groups	2	127.433	3.58675 Va	69.07	0.00978±0.00294
	among populations within regional groups	5	21.941	0.47015 Vb	9.05	0.00000±0.00000
	within populations	50	56.815	1.13631 Vc	21.88	0.00000±0.00000
Margin v. Rise	among regional groups	1	64.726	2.88919 Va	64.14	0.04106±0.00536
	among populations within regional groups	5	21.941	0.46869 Vb	10.41	0.00000±0.00000
	within populations	42	48.149	1.14640 Vc	25.45	0.00000±0.00000
NE v. SW CR	among regional groups	1	7.899	0.44418 Va	35.09	0.07722±0.01012
	among populations within regional groups	3	3.043	0.03277 Vb	2.59	0.21799±0.01260
	within populations	29	22.881	0.78900 Vc	62.32	0.00000±0.00000

### Genetic Analysis

DNA sequences were edited and aligned (using CLUSTAL-W) in Geneious Pro 5.3.4 [50]. Bi-directional sequences were used with the exception of one *H. longibranchiata* individual from 7A07 for which only one direction was usable for the*16S* sequence. Final datasets consisted of 680 basepairs of *16S* for *H. longibranchiata*, 524 basepairs of *COI* for *H. longibranchiata*, and 526 basepairs of *COI* for *M. gracilis*. DNA sequences have been deposited in GenBank (JX219896 - JX219956, *H. longibranchiata 16S*; JX219786 - JX219843, *H. longibranchiata COI*; and JX219844 - JX219895, *M. gracilis COI*). Sequences (and subsequent species identity) were compared to the Genbank database (http://www.ncbi.nlm.nih.gov/) and alignments of *COI* were translated into amino acid sequences and checked for stop codons to assess whether or not the amplified fragments could be considered pseudo-genes. The genetic distances among individuals in each dataset were compared to genetic distances among species within the same families and/or genera to correlate genetic divergence and morphological species boundaries (i.e., to assess the possibility of cryptic species). Genetic distances were calculated (using Kimura 2 Parameter model in PAUP) among the *COI* sequences from seven *Munida* species: *Munida spilota*, *Munida stia*, *Munida notata*, *Munida tyche*, *Munida zebra*, *Munida taenia*, and *Munida thoe* [51]. Genetic distances were calculated (using Kimura 2 Parameter model in PAUP) among the *COI* sequences from seven species of onuphid worms: *Diopatra* cf. *ornate*, *Diopatra dentate*, *Diopatra dentate*, *Hyalinoecia sp.*, *Onuphis elegans*, *Onuphis* cf. *iridescens*, and *Paradiopatra quadricuspis* [49].

**Figure 7:**
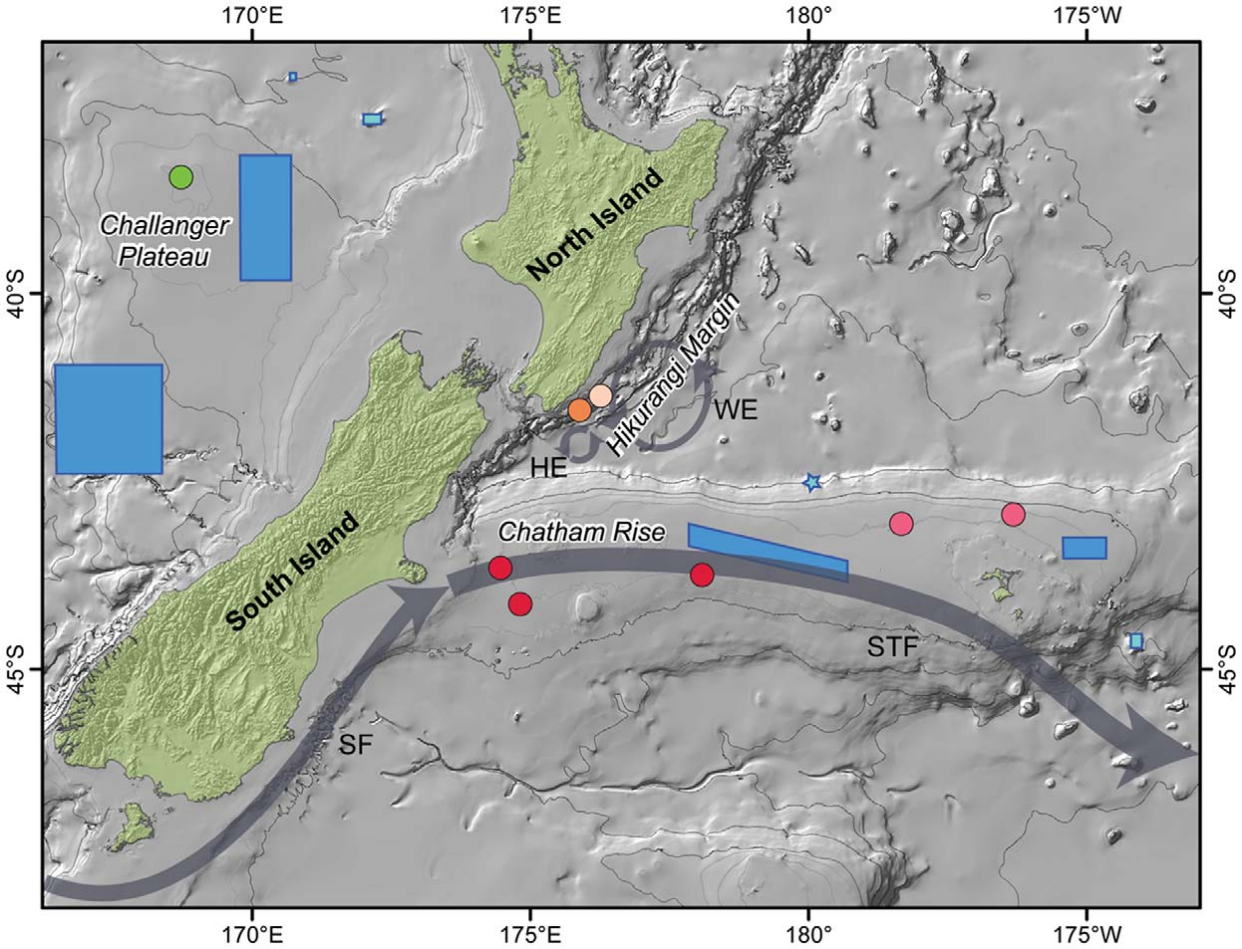
Map of the study area showing genetically distinct populations (colored circles) of the worm *H. longibranchiata* relative to the position of Benthic Protection Areas (blue) and seamount closures (light blue), and local currents. Populations on the Challenger Plateau, Hikurangi Margin and Chatham Rise are green, orange, and red, respectively, with different shades of the latter two colors representing within region differences in genetic population structure. Blue rectangles represent Benthic Protection Areas and Seamount Closures (in light blue). The star marks two Seamount Closures too small to be visible on the map. The approximate position of the Southland Front (SF), the Sub-Tropical Front (STF), the Hikurangi Eddy (HE), and Wairarapa Eddy (WE) are shown with grey bands and arrows. The location of the STF is based on Figure 1 of Hayward *et al.* (2008) [76].

Genetic diversity indices (the number of polymorphic sites in the sequence, the number of haplotypes represented at each site, the haplotype diversity, and the nucleotide diversity) for each population and gene were calculated in DnaSP, version 5.10.01 [52]. In order to test for the significance of population genetic divergences, a measure of population pairwise divergence, or Fst, was calculated with 110 replicates in Arlequin, version 3.11 [53]. In addition to geographically mapping the distribution of haplotypes, we constructed haplotype networks in TCS 1.21 using default program settings [54] to identify potential biogeographic patterns among populations and habitats.

In order to assess population genetic structure among and within populations, Analysis of Molecular Variance (AMOVA) was conducted in Arlequin version 3.11 by grouping sites into the three regions: Chatham Rise, Hikurangi Margin, and Challenger Plateau and running a standard AMOVA with default program settings. To address the question of spatial variation along the Chatham Rise, an AMOVA was conducted to separate the sites into two groups: a northeast subset that consisted of 6C63 and 3CX2 and a southwest subset that consisted of 7A07, 9D28 and 1B15. AMOVA tests were run only on *H. longibranchiata* given that initial assessments indicated there was no genetic structure for *M. gracilis*.

## Results

### Genetic Diversity Indices

Prior to any population genetic analyses, the assessment and confirmation of phylogenetic species was conducted across all sequences for each of the target taxa. The genetic distances among *COI* sequences for various *Munida* species (listed in the Methods section) ranged from 8.2% to 15.4%, while the genetic distances of our *COI* dataset for *Munida gracilis* ranged from 0% to 1.9%. The genetic distances among *COI* sequences for various onuphid worms ranged from 4.6% to 28.5%. Genetic divergences in the *COI* dataset for *Hyalinoecia longibranchiata* ranged from 0% to 2.9%. These results indicate that the individuals we used from each respective morphospecies are within the genetic divergence diagnostic of their respective species, implying that there are no cryptic species in our samples.

A total of 58 *COI* and 61 *16S* partial sequences from *H. longibranchiata* were obtained. There were eight *H. longibranchiata* specimens for which we did not obtain *COI* sequences, but did obtain *16S* sequences. There were four *H. longibranchiata* specimens for which we did not obtain *16S* sequences but we did obtain *COI* sequences. A total of 52 *COI* sequences from *M. gracilis* were obtained. All sequence reads were unambiguous except for one gene sequence for *H. longibranchiata*, which had a single undetermined base. The lack of complete overlap between sequenced individuals for each gene prevented joint analysis of the two genetic datasets. The per-site number of sequences and genetic diversity indices for each gene in both species is presented in Table 2, Table 3, and Table 4.

### Geographic Distribution of Haplotypes

The squat lobster, *M. gracilis* (n = 52), had high haplotype diversity for the *COI* gene (Table 2). Of the 36 haplotypes, only three were shared and the remaining 33 were unique. Two of the three shared haplotypes were found across the Challenger Plateau and the Chatham Rise and the third was absent on the Challenger Plateau but present on the Chatham Rise (Figure 2).

The *COI* sequence dataset for *H. longibranchiata* (n = 58) consisted of 10 haplotypes shared by more than one individual and 10 unique haplotypes for a total of 20 haplotypes (Figure 3). The *16S* sequence dataset for *H. longibranchiata* (n = 61) consisted of nine haplotypes shared by more than one individual and 10 unique haplotypes, for a total of 19 haplotypes (Figure 4). All the haplotypes found at the Challenger Plateau site were unique to that population. There were two haplotypes only found at the Hikurangi Margin sites. Three shared haplotypes were only found on the Chatham Rise, spanning the length of the Rise.

### Haplotype Networks

The *COI* haplotype network for *M. gracilis* reflects the large number of haplotypes, many of which are separated by one or a few nucleotide changes (Figure 5). There is no clear ancestral haplotype, and no significant geographic pattern to the network. This is consistent with the high level of sequence diversity of the *COI* gene of *M. gracilis*.

The *COI* and *16S* haplotype networks (Figure 6) for *H. longibranchiata* are consistent with the geographic structure indicated by the haplotype maps. There appears to be a central, ancestral *16S* haplotype that is present across the Chatham Rise sites, radiating out to the Hikurangi Margin and on to the Challenger Plateau. The *COI* haplotype sequences were more diverse than the *16S* sequences, consistent with common rates of mutation in these genes (e.g., [55]).

### Population Structure

Fst data indicated no genetic structure in the sampled *M. gracilis* populations. Pairwise population Fst values were all below 0.1 and none of the p values showed significant difference among the populations (Table 5). In the sampled *H. longibranchiata* populations, pairwise population Fst values for *16S* indicated that the population at the Challenger Plateau site was significantly different (p<0.05) than all other populations (Fst >0.564), as were both populations from the Hikurangi Margin sites (Fst >0.484), which were also different from one another (Fst  = 0.47989). Differences between populations within the Chatham Rise were small (<0.17647) and only the difference between the two northeast populations at 6C63 and 3CX2 was statistically significant (Table 6).

The *COI* results for *H. longibranchiata* revealed similar trends to the *16S* data, however, there were significant differences between some Chatham Rise populations (Fst >0.31089) (Table 7). Specifically, populations in the northeast were significantly different from those in the southwest and south central part of the Rise.

### Analysis of Molecular Variance

*Hyalinoecia longibranchiata* AMOVA results (Table 8 and Table 9) were consistent with our other analyses. The AMOVA for the three regions revealed that there was higher variation between regions than within regions, indicating that there is significant genetic structure across the study area. The Hikurangi Margin sites were shown to be statistically different from the Chatham Rise sites. The AMOVA tests for differences between groups of sites on the southwest and northeast of the Chatham Rise revealed a greater diversity in population structure within groups than between groups, indicating that this test does not show significant structure based on the northeast-southwest divide.

## Discussion

Our study is one of few that have examined genetic connectivity of deep-sea invertebrate populations in the New Zealand EEZ. Using mitochondrial *COI* and *16S* genes as genetic markers, we tested for genetic structure among populations of the squat lobster *Munida gracilis* and the quill worm *Hyalinoecia longibranchiata* at sites across three deep-sea regions near New Zealand: the Chatham Rise, Hikurangi Margin, and Challenger Plateau. The study aimed to address a number of questions related to the factors that determine genetic connectivity in the deep sea as well as inform the design and evaluation of MPAs in the deep sea. Our results are discussed below in relation to each of these study questions (paraphrased below).

### Is there Regional Genetic Structure Across the Study Area?

The population structure on a regional scale for *H. longibranchiata* provides evidence of little to no historic gene flow between the Challenger Plateau, the Hikurangi Margin, and the Chatham Rise. In contrast, the sampled *M. gracilis* population data demonstrated high haplotype diversity for *COI* and no population structure at the geographic scale examined in this study. A number of factors are known to affect genetic connectivity including large and small-scale current regimes, topography, settlement habitat, depth, dispersal strategies, adult mobility and reproductive success, etc. [56]. While it was not our goal to isolate a single factor as the cause of an observed population genetic structure, we can examine the consistency and potential interplay of each factor in relation to the results.

The observed large-scale genetic differences in *H. longibranchiata* populations between the three regions can be explained partly by geographic distribution and partly by currents. Large geographic distance between sites can limit connectivity, especially for species with low dispersal capability. The Hikurangi Eddy, located to the East of Cook Strait could create an isolated water mass around the study sites at the southern end of the Hikurangi Margin [57], and limit dispersal. There is one shared haplotype between the Rise and one of the sites on the margin, suggesting that historically there has been some ability of individuals to disperse between the two regions. Variation in the spatial extent of the Hikurangi Eddy could transport larvae or adults between the Margin and the Rise. The lack of any shared haplotypes between the Chatham Rise/Hikurangi Margin and the Challenger Plateau is consistent with the Cook Strait functioning as a barrier to dispersal, rather than a conduit for transporting larvae or adults between the western and eastern side of New Zealand. Barnes (1985) [58] found that, despite large tidal flow, a front exists in Cook Strait with up to a 2°C gradient that causes negligible net flow–at least near the sea surface–through the Strait.

In contrast to the quill worm, we found unstructured yet genetically diverse populations of the squat lobster *M. gracilis* throughout the study area. Based on the large proportion of unique haplotypes, we conclude that the genetic diversity of *COI* in the *M. gracilis* population has not been fully ascertained. Considering the high levels of diversity in the mitochondrial genes we sampled, it is difficult to draw conclusions about the *M. gracilis* population except to say that the presence of certain haplotypes across the study area indicates that there is likely a single population with high levels of mixing not impeded by geographic distance or current patterns.

As with all population genetic studies, the numbers of individuals, loci, and sites have to be considered in the interpretation of the data. The available sample sizes at various sites in the study area (which we have termed populations) are not considered large, nor are they consistent across sites. However, this is not atypical for deep-sea population genetic studies in which collecting large sample sizes yielding highly robust estimates of genetic diversity is considerably difficult given the inaccessibility and expense of obtaining these populations. Comparing haplotype diversity between sites with highly variable sample sizes could lead to inappropriate assumptions about spatial patterns of haplotype diversity, including the performance of unbiased estimators and rarefaction methods [59], with lower sample sizes likely underestimating levels of diversity. We calculated Fst from haplotype-frequencies and pairwise DNA sequence diversity. Haplotype frequency-based statistics are more sensitive for small sample sizes, while the sequence-based statistic can be considered a more sensitive method for detecting population structure in highly polymorphic loci. Our goal was not to examine the effect of small or variable sample sizes on genetic estimates in genetically diverse datasets (as in [59]), and we caution the over-interpretation of our results. Given the high COI haplotype diversity of *in M. gracilis*, it is likely that more individuals would provide more informative results. Despite the limited *H. longibranchiata* data set, the data provide insight into the population structure of this species, and the results are supported by both genetic markers (*16S* and *COI*). From our initial genetic survey and for future studies of these species, the *COI* gene can be considered a useful marker for resolving genetic structure in *H. longibranchiata*, and somewhat less in *M. gracilis*.

### Is there Genetic Structure Within the Three Regions?

In addition to the larger scale patterns discussed above, population structure for *H. longibranchiata* was observed between the seamount and slope sites on the Hikurangi Margin and potential differentiation was detected between populations on the northeast and southwest sites on the Chatham Rise. No genetic structure between sites of varying habitat types–specifically between seamount and slope–was observed for *M. gracilis* in any region.

Addressing questions of genetic connectivity is especially complex in the deep sea given that suitable habitats can be patchy over large spatial scales (hundreds to thousands of km). For example, several thousand kilometers may separate hydrothermal vent fields or seamounts and yet gene flow may occur between the geographically distant sites of the same habitat type (e.g., [60], [61], [62], [63]). The opposite can also be true where small distances between patches of the same habitat do not necessarily translate into genetic connectivity among populations if there are physical or biological barriers to dispersal [60].

An added layer of complexity to deep-sea connectivity is the potential for inter-habitat connectivity when different habitats may be found in a small geographic area. For example, in the Norfolk Ridge seamount system, populations on the seamounts have been shown to be genetically connected to populations on the island slope [64]. Conversely, populations at different habitats may not be well connected when the physical or biological attributes of one habitat serve to isolate it from other suitable habitats (e.g., the presence of local isolating hydrographic features as seen in some seamount systems [65]).

Because of the constrained sample availability, the sites from which we were able to obtain samples and the sample sizes did not provide a robust enough dataset to fully understand the extent of inter-habitat genetic connectivity. Nevertheless, our data provide some interesting indications about inter-habitat connectivity. While populations of *H. longibrachiata* at the Hikurangi Margin sites were significantly different from the Challenger Plateau sites and the Chatham Rise sites, they were also significantly different from each other. These sites–one a seamount, the other a slope–are only ∼38 km apart, but are separated by a small canyon. It is possible that there is a local current regime on this margin that limits the connectivity of these two populations or perhaps some habitat preference that results in the observed difference in genetic structure. The seamount in question may likely be considered too small for localized isolating hydrographic features. So alternatively it could be the predominance of down slope currents associated with canyons rather than along slope currents on this margin that are responsible for the limited connectivity between the populations of quill worms at the seamount and slope sites. For *M. gracilis*, the two seamount sites in the Andes Seamount cluster–Diamondhead Seamount and Iceberg Seamount–shared haplotypes with populations at sites elsewhere on the Chatham Rise, indicating that for this species in the New Zealand region, habitat type may not play a strong role in genetic connectivity.

Results suggest that there is variation in the level of connectivity across the Rise. Other studies have found marked differences in benthic community structure between the northern and southern flanks of the rise, which have been attributed to the different environmental and biological conditions imposed by the location of the STF [66], [67], [68], [69]. It is possible that the currents that maintain the STF present a significant barrier to dispersal of individuals among populations of the same species between northern and southern sites. While the location of our study sites for *H. longibranchiata* did not allow us to separate strict north-south effects from possible east-west effects, we were able to test for northeast to southwest variation in the genetic make-up of populations. *COI*, but not *16S*, data provide some support for this hypothesis for *H. longibrachiata* because populations at sites on the northeast of the rise were sufficiently different from those on the southwest of the rise.

Depth has been shown to play a major role in connectivity within and between deep-sea ocean basins and slopes (e.g., [70]) and seamounts (e.g., [60]). It is worth remembering here that our samples were explicitly chosen to fall within a small range of depths; a method of sample selection that could have resulted in a reduced ability to detect whether there was an effect of the STF on population structure on the rise. Nodder *et al.* (2003) [67] found that the most notable differences in benthic communities between northern and southern sites were evident at greater depths (e.g., sites at 2300 meters). So it is possible that populations located deeper than our study sites (below 746 m) could be less well connected across the rise than our results suggest. It is also possible that shallower populations on the crest of the rise (approximately 200 m) may be well connected with one another within the core of the STF, yet be poorly connected to populations at greater depths.

### Do the Inferred Life History Strategies Correlate with the Observed Patterns of Genetic Connectivity?

The results of our study indicate that the genetic connectivity patterns of the two study species are different. Differences in life history strategy and inferred pelagic larval duration likely explain the difference between observed patterns in the two species. Larval dispersal and adult mobility contribute to making the squat lobster a better potential disperser than the quill worm and correlate directly with the differences in inferred patterns of connectivity. The relationship between life history and population structure across seamounts is well documented in Samadi *et al.* (2006) [64], in which the authors found that species with broad dispersal potential had limited to no population structure while the other species with limited dispersal potential had clear population structure.

### What Implications do the Patterns of Genetic Population Connectivity have for MPA Design and the Efficacy of the Current BPAs?

In 2007, a fishing industry-driven initiative resulted in the creation of seventeen areas within the New Zealand EEZ that were designated Benthic Protection Areas (BPAs). These areas comprise roughly 30% of the EEZ and are closed to bottom trawling, but not to other uses such as mining. Still, they are considered by some [28] to fulfill New Zealand’s dedication to protecting at least 10% of its marine environment [5]. The selection criteria for the BPAs included size, low fishing levels, geometrically simple boundaries, and representativeness of the Marine Environment Classification [28]. The population connectivity of benthic organisms was not directly considered in the design of the BPAs.

A deep-water MPA process is scheduled to commence in 2013 [71], coinciding with a review of the BPAs [28]. To facilitate an effective review of BPAs, the closed seamounts, and the future deep-water protected area design process, the “best available” scientific information concerning the habitats and faunal communities need to be considered, as well as input from “offshore experts” [71]. To date, only a single study has challenged the efficacy of BPA design by demonstrating that BPAs located at alternate sites could be more effective at protecting biodiversity and less costly to the fishing industry [29]. The results of our study provide additional information that can be used to evaluate the placement of BPAs and future deep-water MPAs.

The difference in genetic population structure between the squat lobster and the quill worm confirms that, in terms of connectivity, MPA design should consider the implications of protecting assemblages of species with different life history strategies [72]. The findings of our study for common species with high levels of dispersal (like *M. gracilis*), indicate that populations in different closed areas have historically been well connected, and one can reasonably presume are currently connected. However, for common species with limited dispersal capabilities (like *H. longibranchiata*), our study findings provide a framework with which one can analyze the efficacy of future MPA design. At the broadest level, our main finding is that a species with direct development has pronounced population structure across the Challenger Plateau, Hikurangi Margin, and the Chatham Rise (Figure 7). If the maintenance of genetically distinct populations is considered integral to the goal of protecting biodiversity, then large protected areas that possess isolated populations will help to further that goal. Presently, there are BPAs on both the Chatham Rise and the Challenger Plateau but there are no closed areas on the Hikurangi Margin. Other large areas in the study region may also possess populations similarly genetically isolated by current regimes such as large eddy systems, that could also be considered in the future design of deep-water MPAs.

Our results suggest that on smaller spatial scales within regions, local topography and current regimes may have a profound impact on gene flow, leading to the differentiation of populations at different habitats. Populations at slope and seamount habitat in close proximity on the Hikurangi Margin were shown to host genetically different populations. There are some protected areas in the study area that have been specifically closed to trawling in order to protect the communities on seamounts, in part because of the then perceived isolated nature of seamount fauna [27]. The current BPAs, because of the large size design criterion [28], protect multiple habitats including seamounts and hydrothermal vents that are perceived to represent vulnerable marine ecosystems [73], [74]. As such, any large protected area should afford some protection to any genetically distinct populations found at different habitats within a region.

The results of our within region comparison suggest that the location of BPAs on the Chatham Rise may require revision. The central BPA on the Chatham Rise is located in the middle of the crest of the rise at depths of 300–450 m and the BPA at the eastern end of the rise extends over a depth range of 300–900 m. We do not have any population data from these specific locations but we have shown the potential for genetic variation across the Rise. Given this finding and our understanding about differences in benthic communities on the north and south flank of the Rise (e.g., [67]), and the likelihood that populations are genetically structured by depth (e.g., [60], [36]), the two BPAs arranged along the axis of the Rise at shallow depths may not be sufficient to protect the genetic variation of populations on the Chatham Rise. The Chatham Rise is one of the largest geomorphic features of the New Zealand EEZ with a complex and productive ecosystem [75], yet large areas of its seafloor are subjected to disturbance from bottom trawling, and in the future disturbance from mining for phosphorite nodules is likely. Our results suggest that further protected areas, or a re-positioning of the current BPAs, could be considered to afford greater protection to the benthic biodiversity associated with the Chatham Rise through genetic connectivity.

### Future Directions

Our assessment of the genetic connectivity of two abundant benthic invertebrates found throughout a range of deep-sea habitats in the New Zealand EEZ represents a step towards understanding the spatial structure of benthic communities in the New Zealand region, and informing the future design of deep-water MPAs in the region. However, the study has raised a number of questions about the populations of *H. longibranchiata*. For example, what is the true geographic extent of populations found in the three regions–Challenger Plateau, Hikurangi Margin, and Chatham Rise? Are the Hikurangi Margin haplotypes found along the margin to the south or north? Similarly, are quill worm populations on the central north part of the Chatham Rise unique to these sites or will they resemble populations at the northeastern sites? What about populations at other sites to the west of New Zealand? Is the population of quill worms at the Challenger Plateau site different from the sites at the other study regions simply because it is on the western side of New Zealand or is the plateau in some way isolated? Such questions apply to other invertebrate species with potentially limited dispersal capabilities. Future genetic studies of population connectivity should include a greater range of study species in order to generate information useful for the design of protected areas in the deep sea.
